# Joint Variable Selection and Classification with Immunohistochemical Data

**DOI:** 10.4137/bmi.s2465

**Published:** 2009-07-22

**Authors:** Debashis Ghosh, Ratna Chakrabarti

**Affiliations:** 1 Departments of Statistics and Public Health Sciences, Pennsylvania State University, 514A Wartik Lab, University Park, PA 16802; 2 Department of Molecular Biology and Microbiology, University of Central Florida, 12722 Research Parkway, Orlando, FL 32826

**Keywords:** antibody, LASSO algorithm, L_1_ penalty, tissue microarray, protein Ghosh and Chakrabarti

## Abstract

To determine if candidate cancer biomarkers have utility in a clinical setting, validation using immunohistochemical methods is typically done. Most analyses of such data have not incorporated the multivariate nature of the staining profiles. In this article, we consider modelling such data using recently developed ideas from the machine learning community. In particular, we consider the joint goals of feature selection and classification. We develop estimation procedures for the analysis of immunohistochemical profiles using the least absolute selection and shrinkage operator. These lead to novel and flexible models and algorithms for the analysis of compositional data. The techniques are illustrated using data from a cancer biomarker study.

## Introduction

The development of high-throughput assays such as mass spectrometry and gene expression microarrays has led to the generation of large numbers of candidate biomarkers in current medical research. However, while such results and signatures tend to provide great potential for disease prognosis, translating the discovery into a clinically useful biomarker requires more investigation. An important first step typically is to validate the finding using so-called immunohistochemical staining patterns.

In immunohistochemical studies, staining patterns of the biomarker are measured across a variety of samples. One common way this is done is using a tissue microarray.^1^ In this scheme, the “spots” on the glass slide represent tumor cores from different patients, and an antibody for the protein of interest is applied to the slide. The staining patterns are then correlated with patient characteristics. In most instances, the staining is assessed by a pathologist, who assigns a score on an ordinal scale, with larger values corresponding to higher levels of staining. Note that this structure is quite different from an expression microarray, in which the spots are individual genes and proteins of interest, while what is hybridized to the slide is a single sample.

Typically, an analysis of such data requires the creation of a univariate score measuring staining intensity for each sample. Then the score is associated with clinical outcomes using standard testing and regression methods. For example, if the clinical outcome is binary, one could use a parametric or non-parametric two-sample test for association. Alternatively, one could fit a linear regression of staining intensity on clinical outcome or a logistic regression of the outcome on staining intensity.

As noted by Etzioni et al^2^ the staining of tumor samples to the antibody is not completely homogeneous. What is available for certain scoring systems is a multivariate profile of percent of tumor cells staining at each level of the various scoring categories. As an example, we consider tissue microarray data from a candidate prostate cancer biomarker, LIMK1.^3^ LIMK1 is a dual specificity novel serine/threonine kinase which modulates actin dynamics through inactivation of the actin depolymerizing protein cofilin.

The function of LIMK1 in reorganization of the cytoskeleton has been studied extensively during developmental defects.^4^,^5^ Recently, a role of LIMK1 in progression and invasiveness of breast and prostate cancer has been predicted.^6^,^7^ In this paper, we explore the status of LIMK1 staining in the nucleus and cytoplasm as it relates to aggressiveness of prostate cancer.

In this dataset, there were five staining categories, and 50 samples were profiled for LIMK1 nuclear staining. The data for five randomly chosen observations are given in Table 1. Note that by definition, the percentages within each row must add up to one. Such data are referred to as compositional data.^8^ A major advance by Etzioni et al^2^ was to advance the use of composition data analytic techniques for the analysis of immunohistochemical data. They propose the use of Bayesian inference for the so-called logistic normal distribution for compositional data. However, fitting their model requires customized software that is typically not available to data analysts. In this article, we discuss the model proposed by Etzioni et al^2^ and show that it is in fact equivalent to a particular linear discriminant analysis model. Linear discriminant analysis (LDA), pioneered by Fisher,^9^ has been a popular model in the classification literature. Software for fitting LDA is available in most mainstream statistical packages, such as MINITAB, SAS and Splus/R. Thus, the first aim of the article is to show that one can in fact fit the model of Etzioni et al^2^ using LDA methods. A second goal of the article is to jointly perform classification and feature selection within this class of LDA models. Such an approach would allow for the automated inclusion of informative and exclusion of non-informative categories for discriminating samples. In the context of the tissue microarray example, this means that we want to find which staining categories that are informative for predicting aggressiveness. This will be done using the lasso penalty described initially by Tibshirani,^10^ in conjunction with a model selection strategy.

The structure of this article is as follows. In **Results and Discussion**, we outline the data structures and discuss the logistic normal model formulation of Etzioni et al^2^ for analysis of immunohistochemical profiles and equivalence with LDA. We also describe the lasso algorithm of Tibshirani.^10^ The methods are then applied to the motivating dataset. In the **Methods** section, we describe the optimal scoring algorithm for converting the classification problem of linear discriminant analysis into a regression problem.^11^ This will allow for the fusion of the classification using multivariate staining profiles along with automatic lasso-based selection of staining categories that are informative. In addition, we describe methods for additional covariate adjustment and model selection within this framework.

## Results and Discussion

### Data structures and logistic normal model

We will be assuming that we have data (*D_i_*, **Y***_i_*, **X**), *i* = 1, …, *n*, a random sample from (*D*, **Y**, **X**), where *D* denotes the group status, **Y** ≡ (*Y*_1_, …, *Y_p_*) is a *p*-dimensional staining profile, and *X* is a *q*-dimensional vector of covariates. *D* will take values 0 and 1. We let *s*_1_, …, *s_p_* denote the scores assigned to (*Y*_1_, …, *Y_p_*); typically, we take (*s*_1_, …, *s_p_*) to be (0, …, *p* − 1) or (1, …, *p*). It is assumed that *Y_i_* (*i* = 1, …, *p*) takes values in (0, 1) and that $\sum_{i=1}^{p}Y_{i}=1$. While **Y** is assumed to have all non-zero components, in practice zeroes do exist. We follow the recommendations of Etzioni et al^2^ and add in a random noise term.

## Logistic Normal Distribution for Compositional Profiles

Define the (*p* − 1)-dimensional vector

(1) $$Z\equiv \left\{\text{log}\left(\frac{Y_{1}}{Y.}\right),\ldots ,\text{log}\left(\frac{Y_{p-1}}{Y.}\right)\right\},$$

where $Y.=\sum_{j=1}^{p}Y_{j}$. Note that we have transformed the *p*-dimensional vector **Y** into a (*p* − 1)-dimensional vector **Z** in order to remove the constraint $Y.=\sum_{j=1}^{p}Y_{j}$. In addition, the components of **Z** are nonnegative. Thus, transformed vectors are multivariate measurements on the product space (0, ∞)^(^*^p^* ^− 1)^. The probabilistic model proposed in Etzioni et al^2^ for the analysis of immunohistochemical profiles is to assume that conditional on *D*,

(2) $$Z|D\sim N(\mu_{D},\Sigma ),$$

where *μ_D_* is a (*p* − 1)-dimensional mean vector and **∑** is a covariance matrix. The induced distribution for (*Y*_1_*/Y*, …, *Y_P_/Y*) is referred to as the logistic normal distribution in the compositional data analysis literature.^8^,^12^ While interpretation for the parameters on the transformed scale (i.e. **Z**) is easy, it is harder to interpret on the original scale.

In terms of analyzing immunohistochemical profiles, Etzioni et al^2^ adopted a hierarchical model in which priors were placed on *μ_D_* and **∑**. They then used a Markov Chain Monte Carlo (MCMC) sampling algorithm to sample from the the posterior distribution of *μ_D_*. It was used to construct a 95% credible interval for mean shifts for the log-transformed profile. An easier estimation procedure that does not require implementing a Gibbs sampling algorithm is to fit a linear discriminant analysis model to the transformed data **Z**. This can be performed using virtually any standard statistical software package. The estimated linear discriminants from performing the linear discriminant analysis can be used in several ways. First, they can be used as a visualization method. Second, they can serve as a data-driven summary score on which further analysis can be performed.

One also notes that (2) can be generalized to allow for proportional covariance matrices across populations. This would then necessitate fitting a quadratic linear discriminant analysis model to **Z**.

## Lasso Estimation

Shifting gears, we discuss the Least Absolute Shrinkage and Selection (LASSO) algorithm proposed by Tibshirani.^10^ Suppose we wished to fit the linear regression model: *E*(*U_i_|***Y***_i_*) = *β^T^***Y***_i_*, where (*U*_1_, …, *U_n_*) are continuous variables, and *β* is an unknown *p-*dimensional vector of unknown regression coefficients to be estimated. The LASSO solution is given by

$${\hat{\beta }}_{L}=\text{arg}\text{min}\sum_{i=1}^{n}{\left(U_{i}-\beta^{T}{\text{Y}}_{i}\right)}^{2}+\lambda \sum_{i=1}^{p}\left|\beta_{j}\right|,$$

where λ ≥ 0 is a penalty parameter, and *β_j_* denotes the *j*th component of *β*. Tibshirani^10^ showed that placing an *L*_1_ constraint on the sum of the magnitude of the regression coefficients yielded sparsity in the estimates of *β*. To be specific, for certain values of λ, it is possible for the lasso estimate of *β* to be identically zero. Further details on the numerical algorithm implemented here is given in **Methods**.

## LIMK1 Biomarker Study

Using the procedures described above as well as in the Methods section, we now consider a real-life application. The immunohistochemical data come from a putative prostate cancer biomarker, LIM kinase 1 (LIMK1).^3^ In this study, the expression profile of LIMK1 was determined using a prostate tumor tissue array comprising 50 samples from tumors at different stages of progression. The pool of samples in the array included three uninvolved prostate tissues for comparison. TNM classification of tumors in the TMA indicated that 62% patients had histories of either lymph node or distant metastasis at the time of surgery or biopsy, and 88% of the tumors had Gleason scores of 7 or above. Gleason score (GS) is an aggregate measure of the aggressiveness of the tumor. It is composed of a major and minor Gleason score, each of which is scored on a scale of one to five. We dichotomized Gleason score as less than or greater than or equal to eight.

**Figure 1:**
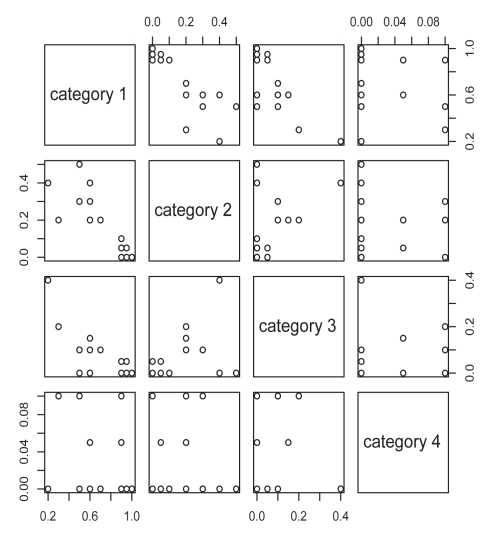
Pairwise plots of nuclear staining by staining category for the LIMK1 study.

**Figure 2:**
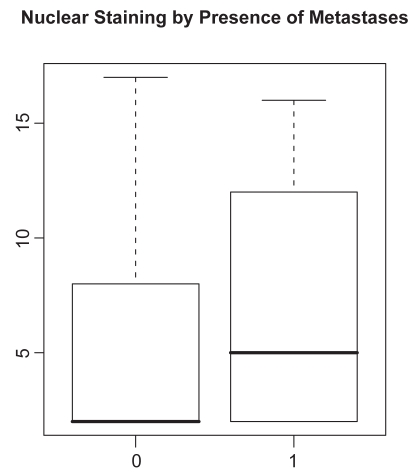
Boxplot of product score for LIMK1 nuclear staining (vertical axis) by presence of metastases (horizontal axis). 0 indicates absence of metastases, while 1 indicates presence of metastases.

**Figure 3:**
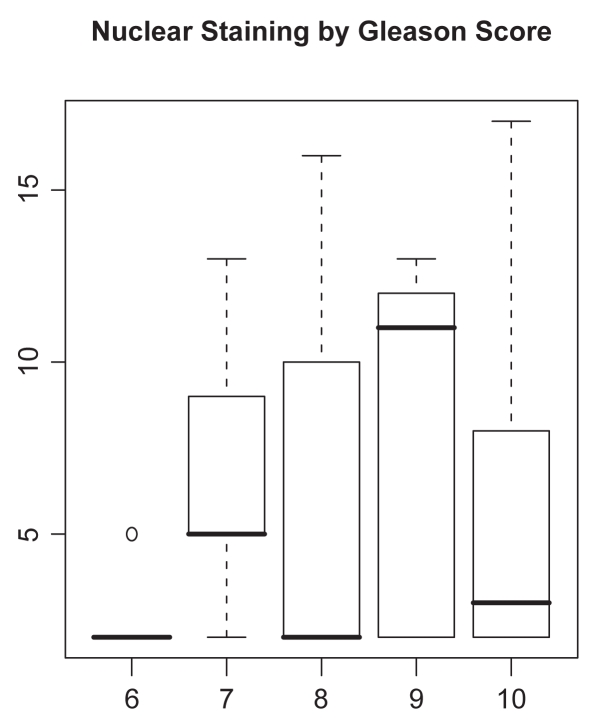
Boxplot of product score for LIMK1 nuclear staining (vertical axis) by Gleason score (horizontal axis).

Analyses of nuclear staining are considered first. Scatterplots of the multivariate nuclear staining profiles by pairwise category comparison are given in Figure 1. To associate staining with the clinical parameters (presence of metastases, Gleason score), we used the product score, multiplying the percentage staining by the staining intensity. Boxplots of the product score for nuclear staining versus presence of metastases and Gleason score are provided in Figures 2 and 3. While Figure 2 indicates that metastatic tumors have higher nuclear staining relative to non-metastatic tumors, there is less difference in nuclear staining across the different Gleason score categories. A t-test reveals the differences corresponding to the boxplot in Figure 2 to be statistically non-significant (*P* = 0.32 for presence of metastases), while an analysis of variance yields the association between nuclear staining and Gleason score to also be nonsignificant (*P* = 0.31).

A series of logistic classification models using the proposed methods were run; they are summarized in Table 1. Based on the analyses we find that if we use nuclear staining profile to predict presence of metastases, then all four staining categories are informative. Using the BIC criterion, there was no improvement by including presence of metastases as a covariate. On the other hand, if one wishes to use nuclear staining to predict Gleason score, then only the third staining category is informative. There is no improvement by including presence of metastases as a covariate.

**Figure 4:**
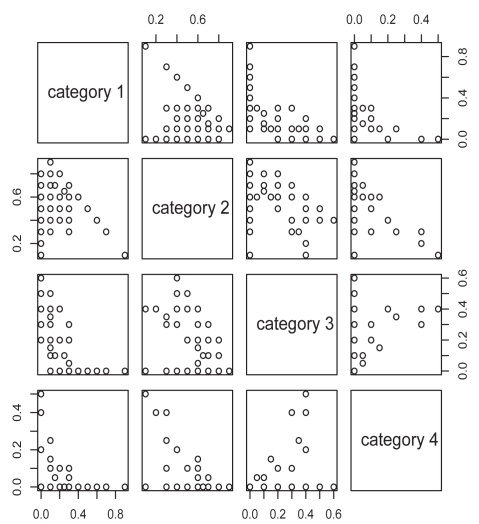
Pairwise plots of cytoplasmic staining by staining category for the LIMK1 study.

**Figure 5:**
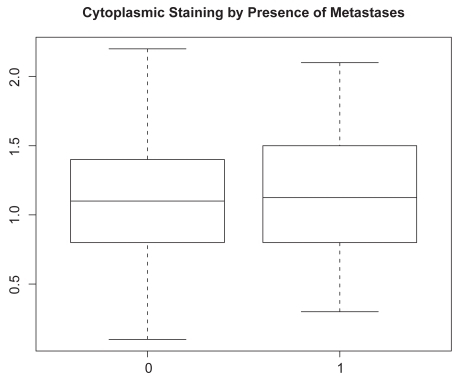
Boxplot of product score for LIMK1 cytoplasmic staining (vertical axis) by presence of metastases (horizontal axis). 0 indicates absence of metastases, while 1 indicates presence of metastases.

**Figure 6:**
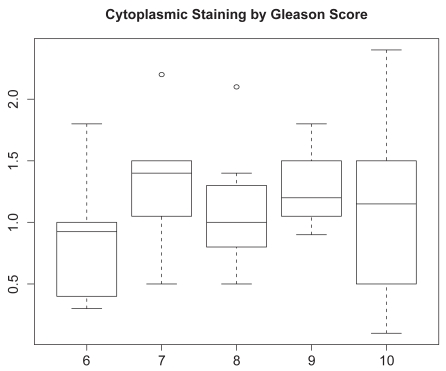
Boxplot of product score for LIMK1 cytoplasmic staining (vertical axis) by Gleason score (horizontal axis).

Next, we considered analyses based on cytoplasmic staining intensity. Pairwise scatter-plots of cytoplasmic staining are given in Figure 4. As with nuclear staining, we used the product score for associating the immunohistochemcial profile for cytoplasmic staining with Gleason score and presence of metastases. The boxplots of the LIMK1 cytoplasmic staining product score by presence of metastases and Gleason status are given in Figures 5 and 6. Analyses analogous to those for nuclear staining reveal nonsignificant associations (*P* = 0.75 and *P* = 0.40 for presence of metastases and Gleason score, respectively).

The logistic normal models results for analysis using the cytoplasmic staining profiles are given in Table 2. For predicting presence of metastases, only the first staining category is needed. However, if we seek to adjust for Gleason score, then categories 1, 3, and 4 are needed. However, the BIC shows that the model fit worsens. If we use cytoplasmic staining to predict Gleason score, then categories 2, 3, and 4 are selected by the LASSO procedure. Including presence of metastases does not improve the model fit. Comparing across the models listed in the Table, we see that models with fewer staining categories selected tend to have better model fit. This supports the use of the LASSO algorithm for automating variable selection while fitting the logistic normal model to the immunohistochemical profiles. This also suggests that the proposed methodology, which in effect can fit a reduced submodel of the model of Etzioni et al^2^ is a better fit to the data rather than the full model of Etzioni et al.^2^

## Conclusions

In this article, we have explored a compositional data model initially proposed by Etzioni et al^2^ that is applicable to the modelling of immunohistochemical biomarker data such as those which might arise from tissue microarrays. We have shown that it has a natural link with linear discriminant analysis, which has been very well-studied in the statistical literature. Consequently, the Etzioni et al model can be fit using standard software packages for LDA, after some data manipulations are performed. The inference we perform is non-Bayesian, in contrast to the Bayesian inference done by Etzioni et al.^2^

We also have developed an automated variable selection procedure within the class of models by incorporating LASSO estimation procedures. The real data example shows that this automated variable selection leads to a better fit. We have also outlined a model selection strategy in which the Etzioni et al model is compared to submodels in which categories are suppressed.

While we dealt with the situation in this paper where **D** is binary (as did Etzioni et al), the optimal scoring algorithm can be easily extended to deal with the case where **D** has more than two levels. One converts **D** into a *n* × (*G* − 1) matrix, where *G* is the number of groups. The regression model that is fit is then a multivariate regression in that the response is multivariate.

Scientifically, while a score-based method such as the product score provides a simple summary statistic for staining data that can then be associated with clinical parameters in tests of hypotheses and regression models, it might oversimplify the data too much. This would be especially undesirable if there is substantial within-sample staining heterogeneity. Thus, methods which explicitly account for the multivariate nature of the staining offer a useful alternative. Compositional data methods are one type of multivariate approach. What our method allows the analyst to do is (1) model the staining profiles in a multivariate manner, (2) incorporate clinical variables as covariates and (3) exclude uninformative staining categories.

## Methods

### Lasso-based optimal scoring algorithm

In this section, we describe our proposal, which entails developing a sparse estimator in the logistic normal model for compositional data. This is done by using the optimal scoring algorithm of Hastie et al^11^ to convert the logistic normal classification problem into a regression problem. This is done in the following way:

1. Choose an initial score matrix **M** satisfying **M**′**C***_P_***M** = **I**, where **C***_p_* = **D**′**D**/*n*, and let **M**_0_ = **DM**.
2. Fit a linear regression model of *M*_0_ on **Z**, yielding fitted values M̂.
3. Obtain the eigenvector matrix Φ of Φ of M′_0_ M̂; the optimal scores are then M = M_0_Φ.

The fitted values obtained at the end of the algorithm are proportional to the linear discriminant analysis coefficients. To extend the algorithm so that we jointly perform classification and automated variable selection, we simply replace step 3 of the algorithm by LASSO estimation of the type described in Results and Discussion. Based on the algorithm, regression coefficients for each variable in **X** will be estimated. Those with estimated regression coefficients that are zero are considered unimportant variables or features.

We will use the algorithm of Osborne et al^13^ for LASSO estimation. Let *σ* be the index set, a subset of {1, …, *p*}. The *i*th component of *β* is non-zero if and only if *I* ∈ *σ*. The algorithm of Osborne et al^13^ operates by sequentially updating the index set. Let *P* denote the permutation matrix that arranges the non-zero components of *η* as the first *s* components, where *s* is the cardinality of *σ*. We have that

$$\beta =P^{T}\left(\begin{array}{c} \beta_{\sigma }\\ 0 \end{array}\right).$$

Let *θ_σ_* be the sign vector of *β_σ_*. At each step of the algorithm, *β* must satisfy the *L*_1_ constraint; this can be expressed as *θ_σ_^T^β_σ_* ≤ *t*. The optimization problem solved by Osborne et al^13^ is to minimize

$$\frac{1}{2}\sum_{i=1}^{n}{\left\{M_{0i}-Z_{i}^{T}(\beta +h)\right\}}^{2}$$

over **h** subject to *θ_σ_^T^*(*β_σ_*+**h***_σ_*) ≤ *t* and

$$h=p^{T}\left(\begin{array}{c} h_{\sigma }\\ 0 \end{array}\right).$$

If the constraint is active, then the optimal solution for **h** is given by the least square method. Let **h** denote the solution, and let (β* = *β* + **h**. If sign(β*)_σ_= *θ*_σ_, then (β* is said to be sign feasible. If (β* is not sign feasible, then the following steps are taken:

1. Find the smallest α ∈ (0, 1) such that 0 = *β_k_* + *αh_k_* for a *k* ∈ *σ* and set *β̃* = *β* +*α* **h**.
2. One of two steps may be taken here. Either
  - Set *θ_k_* = −*θ_k_* and recompute **h**. If *β* + **h** is sign feasible for the revised *θ*, set (β* = *β* + **h** and go to step 3. or
  - Update *σ* by deleting *k*, resetting *β* and *θ_σ_* accordingly, and recompute **h** for the revised problem.
3. Iterate between steps 1 and 2 until a sign feasible *β̃* is obtained. Set β* = *β̃*.

Once the sign feasibility is obtained, the optimality of the candidate solution is tested. This is done by calculating

$$\text{v}=\frac{Z^{T}(D-Z^{T}\beta^{*})}{\left||Z_{\sigma }^{T}(D-Z^{T}\beta^{*})|\right|}=P^{T}\left(\begin{array}{c} {\text{v}}_{1}\\ {\text{v}}_{2} \end{array}\right),$$

where **Z**_σ_ is the design matrix **Z** with columns corresponding to *P*. By definition, the *i*th component of v_1_ is *θ_i_* for 1 ≤ *i* ≤ *s*. If the absolute magnitude of the *i*th component of v_2_ ≤ 1 for 1 ≤ *i* ≤ *(p* − *s*), then β* is a solution to the lasso problem. Otherwise, the following steps are taken:

1. Find the index *j* such that the *j*th component of v_2_ has the largest magnitude.
2. Update *σ* by adding *j* to it and update *β_σ_* by adding a zero as its last element and *β_σ_* by appending the *j*th component of sign(v_2_).
3. Set (β* = *β* and iterate between steps 1 and 2.

This algorithm has been implemented as an R function (www.r-project.org) by the first author and can be obtained upon request.

### Covariate adjustment and model selection

An important question not addressed by Etzioni et al^2^ was adjusting for other co-variates in addition to *D*. Since we have expressed the classification problem as a regression one via the optimal scoring algorithm, we can immediately modify it to account for **X**:

1. Choose an initial score matrix **M** satisfying **M**′**C***_P_***M** = I, where *C^p^* = **D**′**D/***n*, and let M_0_ = **DM.**
2. Fit a linear regression model of **M**_0_ on **X**.
3. Compute the residuals from step 2 and regress on **Z** using the LASSO estimation algorithm, yielding fitted values M̂.
4. Obtain the eigenvector matrix **φ** of M′_0_M̂; the optimal scores are then **M** = **M**_0_Φ.

Notice that this algorithm forces **X** to be in the model so that components of **X** are not set to zero using the LASSO algorithm.

Based on the models, it would be useful to have a criterion for performing model selection. We can do this easily again using the equivalence of the classification and regression problem. We simply use the formula RSS + *p/*2 *log n*, where RSS denotes the residual sum of squares from the linear regression output in the algorithm, and *p* denotes the number of variables that are in the regression model. In particular, variables with estimated zero coefficients are not counted. Lower values of the criterion indicate better model fit. We will refer to this criterion as the Bayesian Information Criterion (BIC), a version of which was proposed by Schwarz.^14^ Note that if the smallest BIC value corresponds to no variables being excluded, then this indicates that the best model fit is that of Etzioni et al.^2^

## Figures and Tables

**Table 1:** Summary of staining data for five randomly chosen observations from prostate cancer data.

Observation	Category 1	Category 2	Category 3	Category 4	Category 5
1	1.000	0.00	0.00	0.000	0
2	0.950	0.05	0.05	0.000	0
3	0.600	0.30	0.10	0.000	0
4	0.600	0.40	0.40	0.000	0
5	0.950	0.05	0.05	0.000	0

**Table 2:** Summary of logistic normal classification models fit using proposed methods in the paper.

Staining Intensity	Group label	Covariate Adjustment	Categories Selected	BIC
Nuclear Staining	Presence of Metastases	None	1,2,3,4	17.41
	Presence of Metastases	Gleason score	1,2,3,4	17.77
	Gleason score	None	3	13.24
	Gleason score	Presence of Metastases	3	16.28
Cytoplasmic Staining	Presence of Metastases	None	1	13.90
	Presence of Metastases	Gleason score	1,3,4	17.77
	Gleason score	None	2,3,4	16.14
	Gleason score	Presence of Metastases	2,3,4	16.28
